# Preparedness of healthcare workers at French Ebola referral centres

**DOI:** 10.1016/j.nmni.2014.12.005

**Published:** 2015-02-17

**Authors:** C. Tarantini, P. Peretti-Watel, Y. Yazdanpana, B. Guery, C. Chidiac, C. Rapp, P. Brouqui

**Affiliations:** 1) IHU Méditerranée Infection, Maladies Infectieuses et Tropicales, CHU Nord APHM, France; 2) INSERM, UMR912 SESSTIM and Southeastern Health Regional Observatory (ORS Paca), Marseille, France; 3) Inserm, IAME, UMR 1137, France; 4) Université Paris Diderot, Sorbonne Paris Cité, France; 5) Maladies Infectieuses et Tropicales Hôpital Bégin, France; 6) Maladies Infectieuses et Tropicales, Hôpital Bichat Claude Bernard, Paris, France; 7) Maladies Infectieuses et Tropicales, HCL–GHN, Hôpital de la Croix Rousse, Lyon, France; 8) Maladies Infectieuses et Tropicales, Hôpital Huriez, Pavillon Fourrier, CHRU de Lille, Université de Lille, France

An epidemic of Ebola haemorrhagic fever (EHF) began in Guinea during December 2013, and the World Health Organization was officially notified on 23 March 2014. Since then, as of 3 December 2014, a total of 17 145 suspected, probable and confirmed EHF cases and 6070 deaths have been reported. Exposure of healthcare workers (HCWs) has resulted in more than 592 of them becoming infected, and at least 340 have died since the start of the outbreak (http://www.who.int/csr/disease/ebola/situation-reports/en/). As of this writing, three hospital-acquired infections have been documented in HCW in Western countries, one in Spain and two in the United States, both in Texas.

In France, two humanitarians were infected with EHF and cared for. Preparedness to treat imported EHF relies on 12 referral centres, nine within French national territory and three in overseas territories. Preparation started after 2001; referral centres were already in use for other crises, including severe acute respiratory syndrome, H1N1 pandemic flu and Middle East respiratory syndrome coronavirus. Training and preparedness at these centres have been evaluated among European centres in several studies [1], [2] (http://epp.eurostat.ec.europa.eu/portal/page/portal/population/data/main_tables). However, EHF poses new problems, as it is the first time that such a highly contagious, untreatable and fatal epidemic disease (class 4 agent) has been imported into Europe. Several previous studies have dealt with the preparation undertaken by referral units and the possibilities of outbreaks of EHF in northern countries [3], [4], [5], but only a few studies address HCWs [3]. We thus evaluated the current feelings of HCWs in France towards their state of preparedness to treat patients with EHF. In order to be fast and up to date, we focussed on only five of the nine referral centres in France (two in Paris and one each in Lille, Lyon and Marseille). One of us (CT) went to meet the HCWs and ask them to fill a short multiple-choice questionnaire, followed by open discussion. After receipt of participant agreement, CT recorded each interview and transcribed them. CT met 83 HCWs (17 auxiliary nurses, 46 nurses and 20 physicians), 47 from infectious disease units (IDUs) and 36 from intensive care units.

Overall, only 48 HCWs (58%) thought that they had received the necessary training to work with patients infected with EHF (74% among the IDU personnel), 53 (64%) felt ready to receive an infected patient in their unit (81% among the IDU personnel) and 60 (73%) wished to be personally involved in their care (83% among the IDU personnel) (Table 1). These results reveal these HCWs’ habits of working under restrictive protocols at a high risk of infection in a sanitary-crisis context in IDUs. Moreover, practice in these units is often the HCW’s personal choice; these HCWs consider it an opportunity to care for patients with rare diseases. However, it is necessary to moderate these results. Firstly, the answers to open questions reflected the gap between feeling prepared and the wish to care for a contagious patient—the latter mainly due to a sense of duty and medical ethics [6], [7] (for example, the duty to provide care despite the risks)—as well as the intellectual and professional stimulation caused by an extraordinary situation with an uncommon disease of which the medical profession has little knowledge. Secondly, the continual evolution of protocols and measures fuels the feeling of unpreparedness, although this continual evolution also justifies the opinion shared by many HCWs that protocols and measures improve each day and that they are on the right track.

There were significant differences in feeling prepared among auxiliary nurses (82%), physicians (80%) and nurses (50%). It is important to note that auxiliary nurses are not involved in intensive care. Physicians are more involved in the protocols process and may consequently be more confident. Nurses are the personnel who provide direct care, while auxiliary nurses are second-line personnel. Our results thus indicate that the most involved and exposed HCWs are also those who feel the least prepared and who report having the least faith in the protocols process. These outcomes may explain why 47 HCWs (58%) said that they felt unsafe concerning the potential transmission of EHF from patient to HCW. Eighty percent of these HCWs were in intensive care units and 42.6% in IDUs. Regarding occupational status, auxiliary nurses were the least likely to feel unsafe (35%), while the rates for nurses (67%) and physicians (60%) were approximately the same. HCW habits according to occupational status probably also play an important role in risk perception, but a more detailed study would be necessary to confirm this hypothesis. We have to take into consideration the fact that all interviewed auxiliary nurses worked in IDUs, where HCWs are used to working under constraining protocols and practicing in situations that place them at high risk of infection, which probably inures them to feelings of risk.

Concerning HCW occupational surveillance during the provision of care for a patient with confirmed EHF, 56% of HCWs thought that surveillance was adequate. Medical and psychological monitoring was thought to be adequate in 60% and 35%, respectively, but 22% and 29% of the HCWs hesitated to answer. If we note a low confidence level regarding HCW occupational surveillance, the high rate of “don’t know” responses clearly reveals the feeling of an important lack of information on this subject.

To conclude, French HCWs, especially auxiliary nurses and nurses, express concerns and doubts regarding EHF-related protocols and measures, but they retain their desire to provide care. We observed differences between units; we also found that the perception of risk is partly dependent on HCWs’ being used to working under constraining protocols and practicing in situations that place them at high risk of infection. Occupation also plays a major role in HCWs’ feelings about the Ebola crisis and the measures adopted. Occupational hierarchy, involvement and exposure influence HCWs’ speech and practices.

## Conflict of interest

None declared.

## Figures and Tables

**Table 1:** Responses to questionnaire administered to HCWs at French Ebola referral healthcare centres

Characteristic	Do you think that you received the necessary preparation and training to practice with a patient infected with EHF?			As a HCW, do you feel ready to receive a patient infected with EHF in your unit?			If a patient infected with EHF arrived in your unit, do you wish to be a part of the healthcare team providing care?			As HCW, do you feel unsafe concerning the infectious risk from EHF-infected patients?		
Yes	No	Don’t know	Yes	No	Don’t know	Yes	No	Don’t know	Yes	No	Don’t know
Overall	48/83 (58%)	33/83 (40%)	2/83 (2%)	53/83 (64%)	24/83 (29%)	6/83 (7%)	60/83 (72%)	15/83 (18%)	8/83 (10%)	49/83 (59%)	34/83 (41%)	0/83 (0%)
Occupation												
Auxiliary nurse	NS	NS	NS	14/17 (82%)	3/17 (18%)	0/17 (0%)	NS	NS	NS	6/17 (35%)	11/17 (65%)	0/17 (0%)
Nurse	NS	NS	NS	23/46 (50%)	19/46 (41%)	4/46 (9%)	NS	NS	NS	31/46 (67%)	15/46 (33%)	0/46 (0%)
Physician	NS	NS	NS	16/20 (80%)	2/20 (10%)	2/20 (10%)	NS	NS	NS	12/20 (60%)	8/20 (40%)	0/20 (0%)
Unit												
Infectious disease	35/47 (75%)	11/47 (23%)	1/47 (2%)	38/47 (81%)	8/47 (17%)	1/47 (2%)	39/47 (83%)	4/47 (8,5%)	4/47 (8,5%)	20/47 (43%)	27/47 (57%)	0/47 (0%)
Intensive care	13/36 (36%)	22/36 (61%)	1/36 (3%)	15/36 (42%)	16/36 (44%)	5/36 (14%)	21/36 (58%)	11/36 (31%)	4/36 (11%)	29/36 (81%)	7/36 (19%)	0/36 (0%)

HCW, healthcare worker; EHF, Ebola haemorrhagic fever; NS, nonsignificant by chi-square test.
